# Supplementary figures and images for: Nintedanib induces gene expression changes in the lung of induced-rheumatoid arthritis–associated interstitial lung disease mice
Source: PLoS One. 2022 Jun 17;17(6):e0270056. doi: 10.1371/journal.pone.0270056 (PMC9205484; doi:10.1371/journal.pone.0270056)

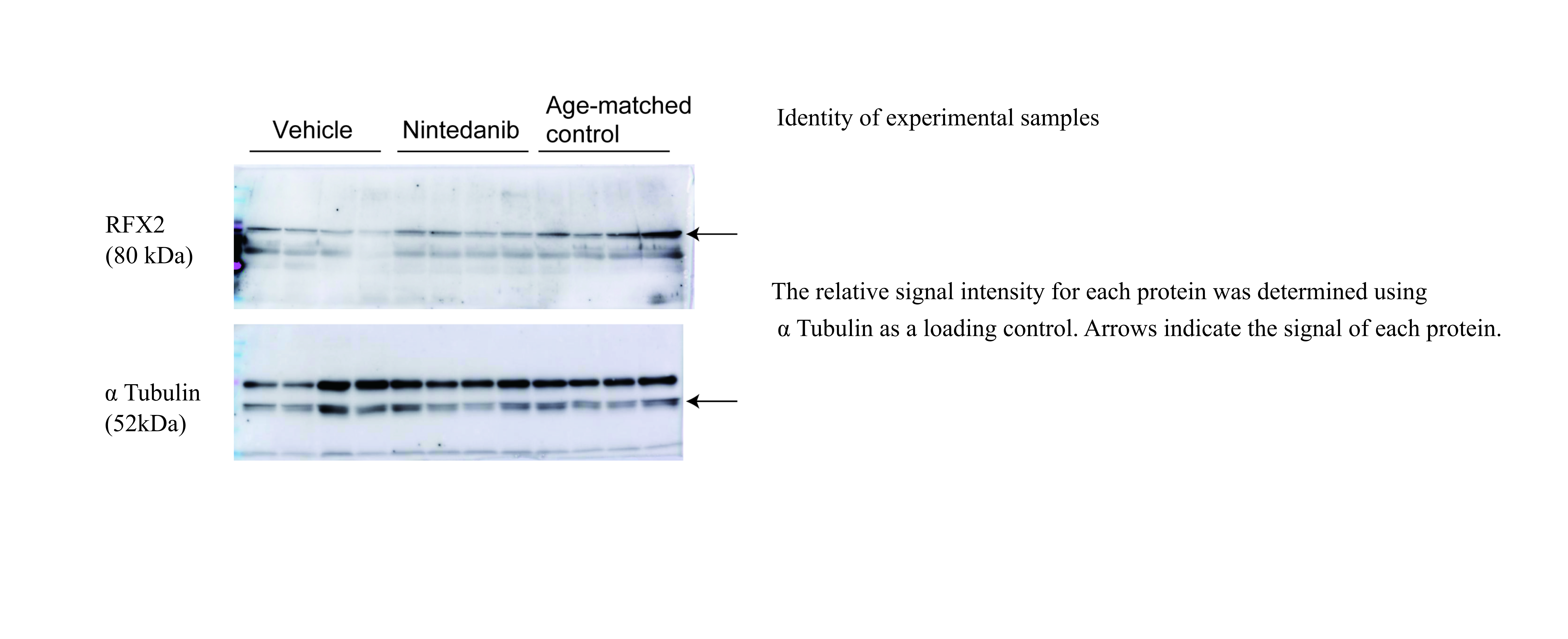

Supplement: S1 Raw images — (TIF) [file pone.0270056.s001.tif]
