# Supplementary material for: Nintedanib induces gene expression changes in the lung of induced-rheumatoid arthritis–associated interstitial lung disease mice
Source: PLoS One. 2022 Jun 17;17(6):e0270056. doi: 10.1371/journal.pone.0270056 (PMC9205484; doi:10.1371/journal.pone.0270056)
Supplement: Attachment — Submitted filename: Response to Reviewers.docx [file pone.0270056.s004.docx]

**Responses to Reviewer 3**

In this study, the authors evaluated the effects of nintedanib on gene expression in the lung of iRA-ILD model mice and revealed that Rfx2 and Bmpr2 were upregulated by nintedanib. But the authors could not confirm the RNA-seq data by RT-qPCR and western blotting using lung tissue.

Major comments

The authors revealed the effects of nintedanib on gene expression in the lung of iRA-ILD model mice and focused on two specific genes, Rfx2 and Bmpr2. They tried to confirm their RNA-seq data by RT-qPCR and western blotting using lung tissue, but there were no statistically significant changes by nintedanib. The authors mentioned nintedanib tended to alleviate the downregulation of Rfx2 and Bmpr2 gene expression or protein expression, but they did not show the p values. It is unclear how many lung tissues the authors used for the confirmation experiments but increasing the number of samples may make a significant difference.

(Response) We thank the reviewer for these insightful suggestions. We added p-values in Figures 4A and C, and Figures 5A and C. We have described the number of samples in the legends of Figures 4 and 5.

RNA-seq results in a small number of cases often cannot be replicable. The author is also necessary to focus on other genes that popped up in RNA-seq data.

(Response) We found 157 up- and downregulated genes by nintedanib treatment. We now evaluate expression of the several genes in fibroblasts. At the start of the study, we anticipated previously reported or downstream genes of the PDGFR, VEGFR, and FGFR signaling pathways; however, expression of these genes was not affected by nintedanib. In this study, we focused on a gene upregulated by nintedanib treatment, *Rfx2*, and showed that RFX2 was expressed mainly in the bronchial epithelium. In addition, a gene downregulated by nintedanib treatment, *Muc5b,* is shown to be expressed in the bronchioles and epithelial cells lining honeycomb cysts [references 44 and 45 in the manuscript]. We speculate that expression changes of these genes by nintedanib treatment may be associated with the differentiation of abnormal cell populations in the peripheral airways for alleviation of pulmonary fibrosis. Recently, Calabrese et al. reported RNA sequencing analysis of aggregates of fibroblasts lined by epithelial cells, which they called “epithelial cell (EC)/fibroblastic foci (FF) sandwich”, in lungs of patients with idiopathic pulmonary fibrosis and primary spontaneous pneumothorax as a control [reference 46]. They extracted EC/FF sandwiches using laser capture microdissection and performed RNA sequencing. They identified 23 upregulated genes in these lesions in idiopathic pulmonary fibrosis compared to those in lungs with primary spontaneous pneumothorax. Furthermore, they selected 14 upregulated genes with log fold-change ≥ 2 out of the 23 upregulated genes. Interestingly, five genes (*Scgb3a1*, *Bpifb1*, *Pigr*, *Muc5B*, and *Krt5*) of these 14 upregulated genes coincided with the downregulated genes by nintedanib treatment in the present study. Despite the differences in diseases, species, and sample preparation, these matches are intriguing and suggest that these genes could function in specific areas leading to the development of pulmonary fibrosis. *Scgb3a1*, *Pigr*, *Bpifb1*, and *Muc5B* are related to secretory and mucin proteins, and immune defenses [reference 46]. *Scgb3a1* is shown to be expressed in secretory cells in idiopathic pulmonary fibrosis [reference 47]. The BPIFB1 protein was reported to be upregulated in the small airway epithelium in cystic fibrosis compared to that in the control [reference 48]. PIGR mediates immunoglobulin A for the immune exclusion of inhaled pathogens in bronchial mucosa [reference 49] We previously reported *Krt5* expression in hyperplastic bronchiolar cells in a mouse model of bleomycin-induced interstitial pneumonia [reference 13]. *Muc5b* is expressed in the small airways and epithelial cells lining honeycomb cysts [references 44, 45]. We speculate that these findings suggest that these genes could be involved in the pathogenesis of pulmonary fibrosis or alleviation of pulmonary fibrosis by nintedanib treatment in specific lesions between the small airways and alveoli. Recent gene expression analyses in these lesions are noteworthy for unraveling the mechanism of the development of pulmonary fibrosis, and data accumulation is expected. Further investigations are needed to clarify the function of these five genes.

We have made the following changes in the revised manuscript:

- Discussion, page 22, line 337-340

“However, functional enrichment and pathway analysis showed that fibrosis-related genes such as *Col1a1*, *Fn1*, and *Acta2* were not enriched in the terms "signaling pathway" and "focal adhesion," which are closely related to these tyrosine kinases,”.

- Discussion, page 25-26, line 410-439

“Recently, Calabrese et al. identified 23 upregulated genes in aggregates of fibroblasts lined by epithelial cells, which they called epithelial cell/fibroblastic foci sandwich, in lungs with idiopathic pulmonary fibrosis compared to those in lungs with primary spontaneous pneumothorax by RNA sequencing analysis [46]. Intriguingly, five downregulated genes (Scgb3a1, Bpifb1, Pigr, Muc5B, and Krt5) by nintedanib treatment in the present study were included in the 14 upregulated genes with log fold-change ≥ 2 in the 23 upregulated genes. Scgb3a1, Pigr, Bpifb1, and Muc5B are related to secretory and mucin proteins, and immune defenses [46]. Scgb3a1 is expressed in secretory cells in idiopathic pulmonary fibrosis [47]. The BPIFB1 protein was reported to be upregulated in the small airway epithelium in cystic fibrosis compared to that in the control [48]. PIGR mediates immunoglobulin A for immune exclusion of inhaled pathogens in the bronchial mucosa [49]. We previously reported Krt5 expression in hyperplastic bronchiolar cells in a mouse model of bleomycin-induced interstitial pneumonia [13]. Muc5B is reported to be expressed in the small airways and epithelial cells lining honeycomb cysts [44, 45]. These findings imply that these genes could be involved in the development of pulmonary fibrosis and the effect of nintedanib in specific lesions between the peripheral airways and alveoli despite the differences in diseases, species, and sample preparation between the present study and Calabrese’s study. Further investigations are needed to clarify the functions of these genes.”

- References

We added references 46-49.

- To unify the term in the text,

Page 25, line 400

“*Mucin5b*” was changed to “*Mucin5B* (*Muc5b*)”.

Page 25, line 403

“*Mucin5b*” was changed to “*Muc5b*”.

- To correct an error,

Page 26, line436

“iRA-ILS” was changed to “iRA-ILD”.

- As requested by the journal office,

Materials and methods, Page 4-5, line 95-99

We added “All mouse experiments were performed according to the rules and regulations of the Fundamental Guidelines for Proper Conduct of Animal Experiments and Related Activities in Academic Research Institutions under the jurisdiction of the Ministry of Education, Culture, Sports, Science and Technology, Japan, and were approved by the Committee on the Ethics of Animal Experiments of Nagoya City University.”
